# Structural insights on the pamoic acid and the 8 kDa domain of DNA polymerase beta complex: Towards the design of higher-affinity inhibitors

**DOI:** 10.1186/1472-6807-8-22

**Published:** 2008-04-16

**Authors:** Corinne Hazan, François Boudsocq, Virginie Gervais, Olivier Saurel, Marion Ciais, Christophe Cazaux, Jerzy Czaplicki, Alain Milon

**Affiliations:** 1 University of Toulouse, UPS; IPBS (Institute of Pharmacology and Structural Biology), 205 route de Narbonne, 31077 Toulouse, France; 2 CNRS, IPBS, UMR5089, Toulouse, France

## Abstract

**Background:**

DNA polymerase beta (pol beta), the error-prone DNA polymerase of single-stranded DNA break repair as well as base excision repair pathways, is overexpressed in several tumors and takes part in chemotherapeutic agent resistance, like that of cisplatin, through translesion synthesis. For this reason pol beta has become a therapeutic target. Several inhibitors have been identified, but none of them presents a sufficient affinity and specificity to become a drug. The fragment-based inhibitor design allows an important improvement in affinity of small molecules. The initial and critical step for setting up the fragment-based strategy consists in the identification and structural characterization of the first fragment bound to the target.

**Results:**

We have performed docking studies of pamoic acid, a 9 micromolar pol beta inhibitor, and found that it binds in a single pocket at the surface of the 8 kDa domain of pol beta. However, docking studies provided five possible conformations for pamoic acid in this site. NMR experiments were performed on the complex to select a single conformation among the five retained. Chemical Shift Mapping data confirmed pamoic acid binding site found by docking while NOESY and saturation transfer experiments provided distances between pairs of protons from the pamoic acid and those of the 8 kDa domain that allowed the identification of the correct conformation.

**Conclusion:**

Combining NMR experiments on the complex with docking results allowed us to build a three-dimensional structural model. This model serves as the starting point for further structural studies aimed at improving the affinity of pamoic acid for binding to DNA polymerase beta.

## Background

DNA polymerase *β *(pol beta) is the smallest human DNA polymerase and the first discovered adaptative polymerase. The structure of the full-length protein of 39 kDa has been solved by X ray cristallography [1]. It is divided into two structural subdomains, which have distinct functions. The first is termed the 8 kDa domain and is located at the N-terminal position. It binds to single-stranded and double stranded DNA, recognizes the 5'-phosphate group in gapped DNA and possesses dRP lyase activity [2]. The second, the C-terminal 31 kDa domain, displays the catalytic activity of polymerisation [3].

Pol beta is the major enzyme of the single-stranded break DNA repair and base excision repair pathways [4-6]. It is able to remove damaged base residues, nucleotides and abasic sites arising from various endogenous and exogenous sources [7]. Thereby, when pol beta gene is deleted from mouse fibroblasts, hypersensitivity to monofunctional alkylation agents, e.g., methylmethanesulfonate, is observed [8,9]. Furthermore, pol beta is able to bypass DNA lesions which block the replication by the replicative DNA polymerases. In fact, pol beta allows an error-prone translesion replication of some adducts, like those generated by cisplatin [10-13]. A large and readily adaptable binding site and a lack of 3'-5'-exonuclease activity facilitates synthesis through lesions. Hence, pol beta seems to be involved in chemotherapeutic agent resistance, as its overexpression diminishes the efficacy of anticancer drug therapies using cisplatin [14]. Indeed, in breast, colon and prostate tumors, a cisplatin resistance is often observed while pol beta is overexpressed [15].

Since it causes genetic instability and resistance to anticancer drugs, pol beta is a therapeutic target. Within the last few years, there has been considerable effort to find inhibitors of pol beta with higher affinity and greater specificity [16-18].

Among the approaches that aim at conceiving inhibitors, the fragment-based drug design is a recent one, that has proven to be successful [19]. Briefly, in this approach, two weakly binding ligands or fragments of the target are sought so that they can be covalently bound to obtain a final molecule, whose affinity is roughly the product of individual affinities of the two fragments [20].

To use this approach, sizeable structural information is required, in particular orientation and conformation of both fragments in their respective sites on the target. This information, together with the distance between fragments, is used to define a linker capable of connecting both fragments without disrupting the global affinity. Nuclear Magnetic Resonance (NMR) spectroscopy is a very powerful structural technique in the fragment-based strategy, as it can detect weakly interacting fragments and provide distances between target and ligand protons and structural information on the interaction. In the context of searching a pol beta inhibitor, we concentrated our efforts on the 8 kDa domain, defined by a protease sensitive hinge region at Lys87. Its structure has been solved by NMR [21,22]. It is formed by 4 alpha helices, packed as two antiparallel pairs. The helices 1 (from Gly13 to Val 29) and 2 (from Ile33 to Lys48) are linked by a 4 amino-acid loop. A "helix-hairpin-helix" motif links helix 3 (from Ser55 to Lys61) to helix 4 (from Thr67 to Thr79), which is responsible for non-specific DNA binding [23,24]. The alpha helix from Arg83 to Gln90 formed in the full-length protein is not folded in the 8 kDa domain. The first step of polymerisation process by pol beta involves the 8 kDa domain. Moreover, the alkylation-sensitive phenotype can be rescued by expression of the 8 kDa domain, suggesting that removal of the dRP group is most critical during base excision repair [25].

Among the large number of pol beta inhibitors that have been reported so far, the pamoic acid binds to the 8 kDa domain with a reasonable affinity [26]. Its solubility in aqueous buffer makes it interesting for further NMR analysis. Moreover, the presence of hydroxyl and carboxyl groups allows simple chemical modifications, aiming at tethering other fragments.

This paper describes structural data obtained on the complex of pamoic acid with the 8 kDa domain. A combination of computational approaches and experimental data obtained from NMR provided the exact orientation of pamoic acid bound to the 8 kDa domain, allowing to propose a three-dimensional model of the complex. We found that pamoic acid binds to a site formed by helix 2 and helix 4, which also corresponds to the single-stranded DNA binding site. The conformation of the bound pamoic acid affords possible binding of a second fragment in its vicinity. The current work is the starting point to apply the fragment-based strategy, that is to say identifying a second fragment that binds in the vicinity of the pamoic acid site.

## Results and Discussion

### Docking pamoic acid to the 8 kDa domain

We used AutoDock 3.0.5 [27] to dock pamoic acid to the 8 kDa domain of pol beta (PDB code 1DK3). Structures generated by AutoDock have been ranked according to their binding energy with the protein and 100 lowest energy structures were retained for further analysis. With the force field used by AutoDock, the energy values for the best ligands varied from -9.58 kcal/mol to -8.96 kcal/mol.

**Figure 1:**
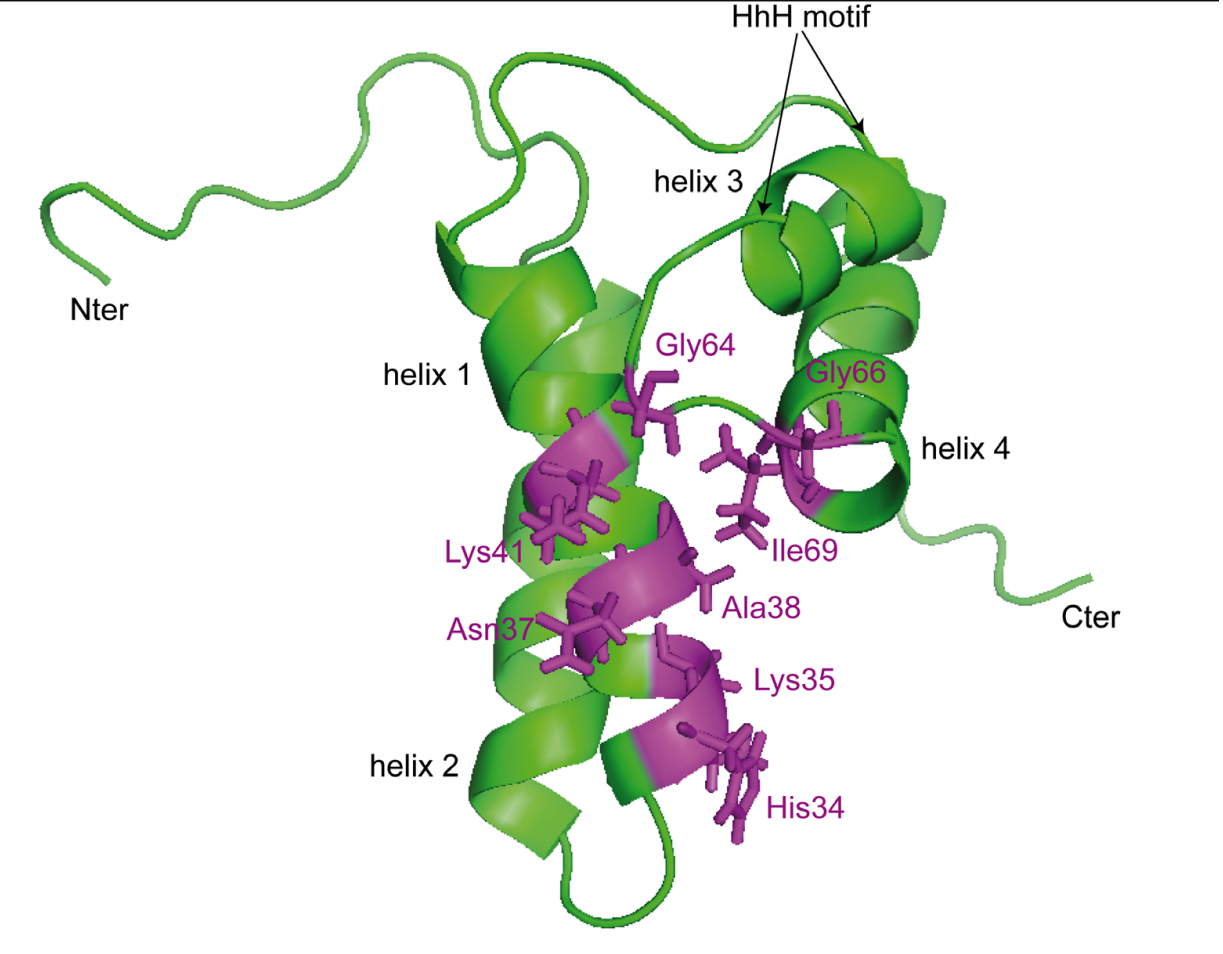
**Mapping residues found close to pamoic acid by docking experiments on the structure of the 8 kDa domain**. Ribbon view of the 3D structure of the 8 kDa domain (PDB code 1DK3), highlighting residues found to be involved in binding with pamoic acid from docking experiments (colored in magenta). The four alpha helices and the "HhH" motif are annotated. Picture was prepared with PyMOL [48].

**Figure 2:**
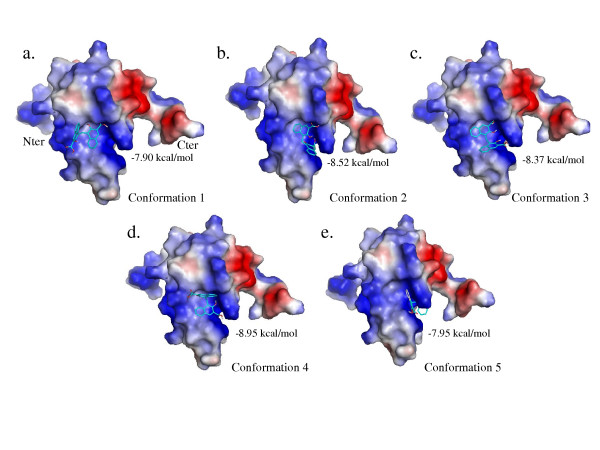
**Docking experiments result in 5 families for pamoic acid conformations in the 8 kDa domain**. The five main conformations of pamoic acid generated by docking experiments are shown against on the electrostatic surface of the 8 kDa domain generated by Pymol. The positively charged surface is colored in blue and the negatively one is colored in red. The orientation is the same as in Figure 1. The best final docked energy calculated by AutoDock appears for each family of conformations. Pictures were prepared with PyMOL [48].

Systematic analysis of the 100 best docked structures revealed that all of them were located in a single site, although pamoic acid could move freely around the 8 kDa domain during docking. Close atomic contacts between pairs of protein-ligand atoms, with a distance cutoff of 2 Angströms, were computed. Nine residues, namely His34, Lys35, Asn37, Ala38, Lys41 on helix 2 and Gly64, Gly66, Lys68, Lys69 on helix 4, were frequently found to be close to pamoic acid. In fact, in more than 50% of the resulting conformations, at least one proton of Ala38, Lys68 and Ile69 was located within 2 Å from pamoic acid. For residues His34, Lys35, Asn37, Lys41, Gly64 and Gly66, over 20% of the 100 best docked structures contained a pair of protein-ligand atoms with a separation below 2 Å. Mapping these residues onto the 8 kDa domain structure indicated that they form a single positively charged groove at the surface of the 8 kDa domain (Fig. 1). Interestingly, Lys35, Lys60 and Lys68, which have been shown to be responsible for single-stranded DNA binding by site-directed mutagenesis [28], are located in the groove where pamoic acid binds to. As this groove is the one where DNA binds, pamoic acid is likely to interfere with single-stranded DNA recognition. Clustering the 100 best ligand structures has been performed using an RMSD (Root Mean Square Deviation) cutoff value of 2 Å. The five resulting clusters indicated that the ligands adopt five different ensembles of conformations in the binding site described above (Fig. 2). Two conformations, n°2 and n°3, were closer to each other than to any other one. The minimum RMSD value between these two conformations was 2.9 Å. Conformation n°5 showed the highest RMSD value with respect to other conformations, from 7.03 Å with conformation n°1 to 8.12 Å with conformation n°4. RMSD values between others pairs of clusters were between 4.5 Å and 6.5 Å.

Two other sites can be defined in the proximity of the pamoic binding site. The first site could be located further beyond Lys35, between helices 1 and 4 and neighboring Leu19, Leu22, Glu26 or Lys72. The other potential site could be related to Lys60, Leu62 and Ala70, further in the direction of Gly64 and Gly66, which belong to pamoic acid site. Since we intend to modify one of the carboxyl groups of pamoic acid to tether a second fragment to reach one of the other two sites, the precise orientation of pamoic acid in the binding site has to be determined.

### Chemical Shift Mapping confirms the pamoic acid binding site found by docking

**Figure 3:**
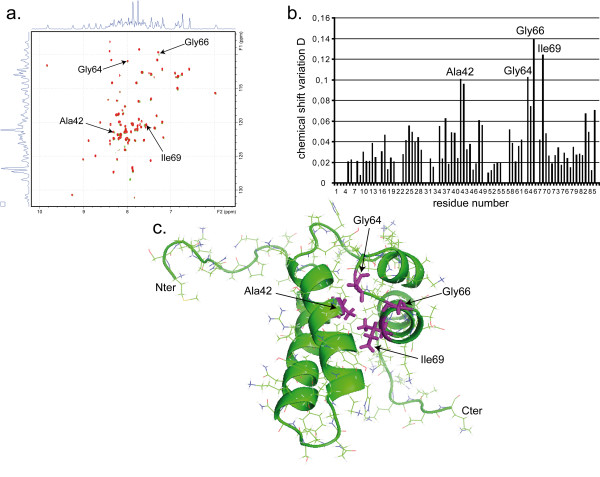
**Chemical Shift Perturbation analysis of the 8 kDa domain upon pamoic acid binding**. **a**. Superposition of 2D ^15^N-^1 ^H HSQC spectra of the ^15^N-labeled 8 kDa domain recorded without (green peaks) and with (red peaks) a 4-fold molar excess of pamoic acid. The four most perturbed residues crosspeaks are annotated. **b**. Histogram of chemical shift variation D upon binding to pamoic acid. Ala42, Gly64, Gly66 and Ile69, which showed a significant D value, higher than 3-times the standard deviation, are indicated. **c**. Mapping of the four residues Ala42, Gly64, Gly66 and Ile69 on the structure of the 8 kDa domain. Sidechains of amino-acids shown by Chemical Shift Mapping to be involved in the interaction are colored in magenta. The orientation of the molecule is the same as that in Figure 1. The picture was prepared with PyMOL [48].

In order to discriminate between the five different conformations, binding of pamoic acid to the 8 kDa domain of pol beta was probed using NMR chemical shift mapping [29] (Fig. 3). This technique is a powerful tool to reveal an interaction between the ^15^N-labeled protein and the unlabeled ligand and shows which residues of the protein are involved in the binding. Therefore ^1^H, ^15^N assignment of protein resonances is a prerequisite. A two dimensional (2D) ^1^H-^15^N HSQC (Heteronuclear Single Quantum Coherence) spectrum of the ^15^N-labeled human 8 kDa domain, corresponding to the first 87 amino-acid residues of pol beta, was recorded. Protein ^1^H and ^15^N resonances were assigned using TOCSY-HSQC and NOESY-HSQC based strategies and confirmed NMR data previously reported for the 8 kDa domain [30]. A satisfactory agreement of HSQC spectra with the reported data insured that the protein is well-folded [31,32].

A series of 2D ^1^H-^15^N HSQC spectra was monitored upon addition of pamoic acid. As the spectra could be easily superimposed, we concluded that the structure of the 8 kDa domain is not altered upon ligand addition. Chemical shifts of the crosspeaks have been followed during titration (Fig. 3a). The peak positions changed between those characteristic of the free and bound forms, indicating fast exchange on the NMR chemical shift timescale.

Comparison of the two spectra recorded for the protein in its free state and in the presence of a 4-fold molar excess of pamoic acid revealed a small number of amino-acids that are affected upon ligand binding. The chemical shift changes D (cf. NMR chemical shift mapping of Methods section) of the same crosspeak between the free and the ligand bound state were plotted *versus *the pol beta residue numbers (Fig. 3b). Amide groups of Ala42, Gly64, Gly66 and Ile69 showed a significant D value (≥ 3 × standard deviation), indicating that these amino-acids belong to the binding site of pamoic acid. Previous studies reported that Lys35, Tyr39, Arg83 and Leu85 were also affected by binding [26]. To characterize binding, we used a threshold for D value equal to 3 × standard deviation instead of 1.5 as in the paper [26]. With the multiplier set to 1.5, Lys35, Arg83 and Leu85 were also found to be affected by binding in our experiments. Interestingly, chemical shift mapping at higher protein concentration revealed that Lys35, Tyr39, Arg83 and Leu85 were also affected by binding (data not shown). However, Arg83 and Leu85 are located far from the other affected residues, on an unstructured loop, which is a part of an alpha helix in the entire 39 kD protein. These residues are therefore unlikely to be involved in the binding of pamoic acid to the entire protein.

Furthermore, comparing the results of the *in silico *docking with those obtained from chemical shift mapping showed a good agreement. All retained docked structures are located in the site revealed by Chemical Shift Mapping experiments, the one which corresponds to the single-stranded DNA binding. Among the five conformations proposed by AutoDock, conformation n°5 could be rejected as it was not close enough to Gly64 and Gly66, which have been shown to be involved in pamoic acid binding. Therefore, at this step, four conformations were retained.

### A NOE correlation helps in determining the correct conformation

In order to further discriminate the right position, we looked for intermolecular NOEs. 3D ^15^N-TOCSY-HSQC (TOtal Correlated SpectroscopY) and 3D ^15^N-NOESY-HSQC (Nuclear Overhauser Enhancement SpectroscopY) spectra of the free ^15^N-labeled 8 kDa domain were acquired and 3D ^15^N-NOESY-HSQC spectrum was recorded in the presence of equimolar pamoic acid concentration.

**Figure 4:**
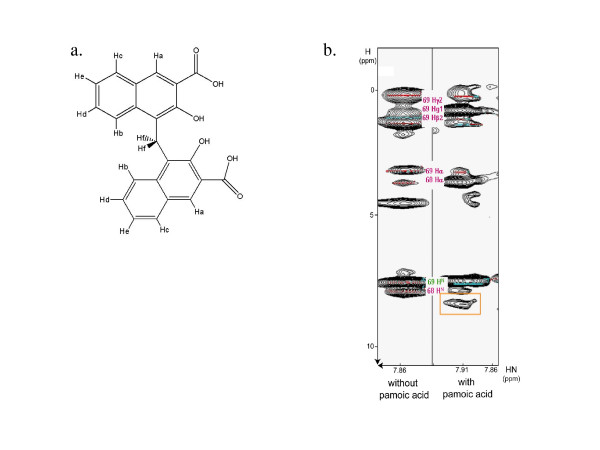
**A NOE correlation indicates a distance lower than 6 Angströms between the Ha proton of pamoic acid and the amide proton of the 8 kDa domain Ile69**. **a**. Structure of pamoic acid with proton symbols, as used in the text. **b**. Two strips of 3D NOESY-HSQC spectrum corresponding to the Ile69 amide proton. The first strip was recorded without pamoic acid and the second in the presence of equimolar pamoic acid concentration. On the second strip, an additional crosspeak, orange-framed, appeared at 8,65 ppm. This crosspeak revealed an intermolecular NOE between the Ha pamoic acid proton and the amide proton of the 8 kDa domain Ile69.

Pamoic acid protons (Fig. 4a) have been assigned in a straightforward manner using COSY-DQF, TOCSY and NOESY experiments (data not shown). Careful analysis of both spectra revealed the presence of an intermolecular NOE correlation peak. The chemical shifts of this NOE corresponded to the amide proton of Ile69 and to the proton Ha of pamoic acid, indicating that these protons are located within 6 Å from each other (Fig. 4b).

The atomic contacts were computed from the four retained structures by assuming that the maximal distance between a pair of protein-ligand atoms does not exceed 6 Å. Among these distances we selected the ones corresponding to the amide proton of Ile69 and the two Ha protons of pamoic acid. The 55 best structures that fulfilled this condition were visually inspected and showed that three different conformations were admissible. More than half of the 55 structures corresponded to conformation n°1 depicted on Fig. 2a. The two other conformations represented 30% (conformation n°2, Fig. 2b) and 18% (conformation n°3, Fig. 2c) of the full AutoDock structure set.

### Two important protons for the interaction between the 8 kDa domain and pamoic acid

In order to get further structural information on the binding, Saturation Transfer Difference (STD) experiments were carried out. The principle of this technique is to transfer the saturation from the protein to the bound ligand [33]. Protein protons that are shifted outside of the spectral window of low molecular weight compounds are selectively irradiated. Within a short time, the saturation is spread over the entire 8 kDa domain through spin diffusion. The spin diffusion is more efficient when the molecular mobility is restricted, which is the case for large molecules like proteins. The spin diffusion may also occur between a protein and its ligand, assuming they are close enough. The closer the distance from the binding site, the larger the saturation, indicating the ligand's binding epitope [34].

**Figure 5:**
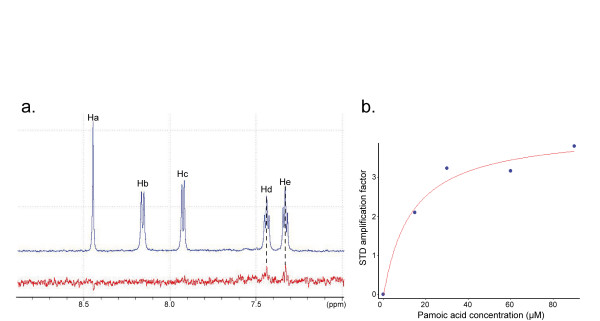
**Saturation Transfer Difference experiments reveal that two pamoic acid protons are essential in the binding to the 8 kDa domain**. **a. **Superposition of STD spectra, without (in blue) and with (in red) selective saturation of the 8 kDa domain protons. Only Hd and He pamoic acid protons revealed binding to the 8 kDa domain. **b**. Plot showing STD amplification factor of He *versus *pamoic acid concentration. Fitted curve is represented in red, corresponding to K_*d *_value of 13 *μ*M ± 5 *μ*M.

We performed STD experiments with the 8 kDa domain at 5 *μ*M and pamoic acid in a 20-fold molar excess. Two pamoic acid protons were found to carry most of the saturation, namely Hd and He (Fig. 4a and 5a), indicating a close proximity with the 8 kDa domain. However, He carried a larger saturation, indicating a closer contact with the 8 kDa domain than Hd [34]. This structural information was used to select the correct conformation of the bound pamoic acid among the three remaining ones.

Searching through the computed atomic contacts, we analyzed the distances between Hd or He and protein atoms and found that two of the three remaining conformations did not feature sufficiently close contacts to give rise to STD signals, namely conformations n°1 and n°3. Moreover, ligand protons that are closer to the protein than Hd and He in these conformations did not give STD signals. By contrast, every structure in conformation n°2 family showed at least one distance below 2,6 Å between these two protons of pamoic acid and one of the protein protons.

STD experiments were used to assess ligand affinity, as the ligand has been added gradually up to around a 50-fold molar excess with respect to the protein concentration. As STD technique requires low concentrations in ligand and protein, it is less affected by interferences due to additional non-specific binding visible in Kd measurements [35]. STD amplification factor was plotted *versus *the ligand excess. Assuming a stoichiometric complex, fitting the curve of the STD amplification factor of the major proton He provided the Kd value of 13 *μ*M ± 5 *μ*M (Fig. 5b) [34].

### Model of the interaction between the pamoic acid and the 8 kDa domain

**Figure 6:**
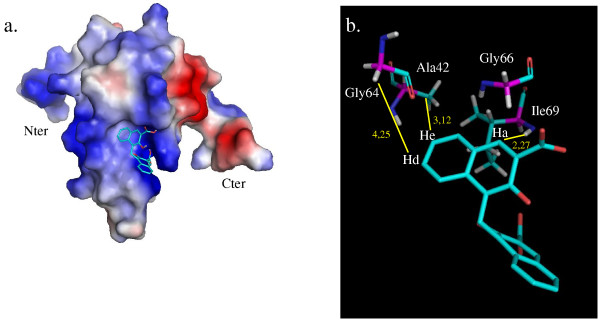
**3D model of the 3D structure of the 8 kDa domain – pamoic acid complex validated by NMR data**. **a**. Electrostatic surface of the 8 kDa domain, with the same orientation as that in Figure 1, illustrating the cavity with the pamoic acid binding site. Blue-colored surface represents positively charged residues and red-colored surface represents the negative ones. Carbon atoms of pamoic acid are colored in cyan and oxygen atoms are colored in red. **b**. Detailed view of the interaction illustrating the binding of pamoic acid to the 8 kDa domain in its experimentally validated conformation. The distances that allowed the identification of the right conformation are shown. The distance between the amide proton of Ile69 and the pamoic acid Ha proton is 2,27 Å. He and Hd are located at 3,12 Å of Ala42 amide proton and at 4,25 Å of Gly64 HA proton, respectively. The pictures were prepared with PyMOL [48].

In the final stage of the analysis, conformation n°2 was retained to establish the model of the complex between the 8 kDa domain and pamoic acid, since it complied with all the NMR data. This structural set of AutoDock results represents an experimentally validated and unique strutural model for the complex 8 kDa domain – pamoic acid (Fig. 6).

Under these assumptions, we analysed the contacts between the validated ligand structure and the 8 kDa domain atoms. The aromatic groups of pamoic acid probably form favorable hydrophobic interactions with the main amino-acids of the site, such as Tyr39, Ala42, Gly64 and Gly66. Amide protons of Gly64 and Gly66 are shielded upon pamoic acid addition. This can be correlated with the fact that both residues are affected by magnetic anisotropy of the naphtalene nucleus [36]. Furthermore, numerous lysine residues present in the site can form electrostatic interactions with both carboxyl groups. One of the carboxyl groups is oriented towards His34 and Lys35. It makes close contacts with Ile69 amide proton and electrostatic interaction with the terminal ${\text{NH}}_{3}^{+}$ group of the Lys68 sidechain. The other carboxyl group forms hydrogen bonds with the amide proton of Lys68 (distance of 1,67 Å) and with the hydroxyl group of Thr67 (distance of 1,94 Å). Obviously, the two carboxyl groups contribute to pamoic acid affinity for the 8 kDa domain. Using one of them to tether a second fragment is likely to lower the affinity but this may be compensated by the properties of the second fragment. From our data, we have defined two other potential sites close to the pamoic acid binding site (see above). In the proposed model, each of the carboxyl groups is oriented towards one of the other sites. Therefore the possibility of increasing the pamoic acid affinity by using the fragment-based approach could be considered.

## Conclusion

Pol beta gets involved in DNA repair pathway and in translesion synthesis, particularly when it is overexpressed in cancer cell lines treated by cisplatin agent. This process leads to a chemotherapeutic drug resistance, which could be prevented by an adjuvant treatment, that is to say a pol beta inhibitor. One of the key benchmarks for a small molecule to become a drug is the affinity for its target. No currently known pol beta inhibitors rise above micromolar affinity, which is insufficient for any pharmacological development. The X family DNA polymerases is the only one to feature the 8 kDa domain [37]. Hence, an inhibitor of this domain is less subject to bind to replicative DNA polymerases. Moreover, inhibition of the 8 kDa of pol lambda and pol mu, both involved in non-homologous end joining of DNA break [38,39], could improve the radiosensitivity of tumors by preventing cells from repairing radiotherapy-induced DNA damage.

Even if pamoic acid is one of the most known pol beta specific inhibitors, its affinity (low micromolar) has to be improved. Structural insights in the interaction between 8 kDa domain of pol beta and pamoic acid are prerequisites to improve the ligand affinity by applying the fragment-based strategy.

A previous work has reported the binding of pamoic acid to pol beta using chemical shift mapping. Pamoic acid is one of the best known pol beta specific inhibitors. It inhibits the deoxyribose phosphate lyase activity and increases sensibility to MMS [26]. As pol beta has been shown to be a pharmacological target, increasing the affinity of pamoic acid for pol beta could transform pamoic acid into a drug-candidate. In the present paper, we have combined NMR (chemical shift mapping, STD and NOESY data) and computational approaches to generate a detailed 3D model of the complex of the 8 kDa domain of the DNA polymerase with pamoic acid. Validation of the computational model by experimental NMR data gave a unique structure for the complex (Fig. 6). The site occupied by pamoic acid corresponds to the one where single-stranded DNA binds to.

Indeed, the model thus established is the starting point to search for a fragment that could bind in one of the other two sites found in the vicinity of the pamoic acid binding site. The orientation of its bound carboxyl groups towards two distinct potential second sites makes pamoic acid a very interesting candidate for further attempts to increase its affinity for pol beta, using fragment-based approach. Furthermore, as NMR techniques can screen small molecules, they can be used to find a second fragment which binds to the 8 kDa domain in a site, which is close to but disjointed from the pamoic acid binding site [40]. The present structure of the complex opens an avenue for the development of new families of specific pol beta inhibitors by the well-established fragment based approach. Glu26 or Lys72. Another potential second site could be related to Lys60, Leu62 and Ala70, further in the direction of Gly64 and Gly66, which belong to the pamoic acid site. be reported elsewhere.

## Methods

### Protein expression and purification

For NMR experiments, the recombinant 8 kDa-domain of DNA polymerase beta (Met1-Lys87) was produced as His-tag fusion protein in a pET28 plasmid (Novagen) in *E. coli *BL21(DE3). Bacteria were grown in LB medium at 37°C to an optical density at 600 nm of 0.8 before induction with 1 mM IPTG during 4 hours, to obtain an unlabeled sample. Isotopically ^15^N-labeled protein was expressed in minimal (M9) medium containing ^15^NH_4_Cl. Proteins were purified using a Ni-NTA column (HiTrap, Amersham) eluted by an imidazole gradient from 100 mM to 500 mM in 5 column volumes, after 8 column volumes at imidazole 100 mM. The protein was eluted at imidazole 250 mM. The protein was then concentrated to 0.5–2 mM in 20 mM deuterated Tris-HCl, pH = 6.8, 200 mM NaCl. The His-tag was cleaved using one unit of thrombin per milligram of protein overnight at 4°C (Novagen).

### Pamoic acid preparation

Pamoic acid was obtained from Sigma-Aldrich. It was dissolved in high-pH Tris-HCl 20 mM, 200 mM NaCl at a concentration of 10 mM. Then, the pH was brought down to 6.9 by addition of the appropriate volume of HCl. The final solution was stored at 4°C.

### NMR spectroscopy

NMR spectra were recorded at 293 K on a Bruker DRX600 spectrometer equipped with a cryoprobe. NMR samples were prepared in 20 mM deuterated Tris-HCl, pH = 6.8, 200 mM NaCl with 10% D_2_O. NMR data were processed using TOPSPIN software (Bruker) and NMRPipe [41] and analyzed using NMRView [42]. Resonance assignment was performed using previously reported BMRB data [43,44] and 3D TOCSY-HSQC and 3D NOESY-HSQC data. Spectra were recorded on the free ^15^N-labeled 8 kDa domain at 500 *μ*M and on the equimolar complex with 1024, 80 and 128 complex points for ^1^H, ^15^N and ^1^H respectively, and 8 scans. The mixing time for TOCSY and NOESY were 60 ms and 200 ms, respectively.

### NMR chemical shift mapping

For NMR titration, 2D ^15^N-HSQC spectra of the ^15^N-labeled 8 kDa domain of pol beta at the concentration of 150 *μ*M were collected at 293 K after incremental addition of pamoic acid (typically 0.25, 0.5, 1, 1.5, 2, 3, 4 molar equivalents of ligand) with 2048 and 256 complex points for ^1^H and ^15^N, respectively, and 4 scans. The NMR sample was diluted by no more than 10% by increasing the pamoic acid concentration.

As the pamoic acid is in fast exchange rate between the free and bound states compared with the chemical shift difference, the observed signal is at a chemical shift corresponding to the weighted average between the chemical shifts of the free and bound states. For each residue, the chemical shift difference was calculated as :

$$D=\sqrt{{\left(\Delta \delta HN\right)}^{2}+{\left(\Delta \delta N/5\right)}^{2}}$$

The factor of 5 appearing for Δ*δ *N corresponds to the spectral widths ratio of ^15^N (25 ppm) and ^1^H (5 ppm) and is commonly used in the literature [45].

### Saturation Transfer Difference experiments

For STD titration, a sample of 3 *μ*M of unlabeled protein in deuterated buffer was prepared. Increasing amounts of pamoic acid were added, in molar ligand/protein excess from 1 to 30.

Protein signals were saturated by forty 50 ms gaussian pulses, with interpulse spacing of 1 ms. The total saturation time was 2 s. Water signal elimination was performed using Watergate W5 sequence [46]. Protein signals were eliminated by a 60 ms T1*ρ *filter with a 5000 Hz excitation. For each ligand concentration, two spectra were recorded with and without protein irradiation. For I_0 _spectrum, no irradiation of the protein occurred. I *std *spectrum is obtained by substraction of spectrum with protein irradiation from the I_0 _spectrum.

For each ligand proton signal, STD amplification factor is calculated as follow :

$$A_{STD}=\frac{I_{std}}{I_{0}}\cdot ligand\;excess$$

where I_0 _and I_*sat *_are the signal intensity of a ligand proton in the spectra without and with protein irradiation, respectively. The dissociation constant was obtained from least-squares fitting of *A*_*max *_as a function of total ligand concentration according to the following equation :

$$A_{STD}=A_{max}\times \frac{\left[L\right]}{[L]+K_{d}}$$

[*L*] represent pamoic acid concentration. *K*_*d *_and *A*_*max *_were searched parameters in fitting using the GOSA program [47].

### Docking experiments

The protein-ligand docking was performed using the AutoDock 3.05 software [27]. The input files were prepared with the MGLTools 1.2. A pdbqs file was created, based on PDB: 1DK3 file information. Polar hydrogens were added and charges were calculated. From these data, a grid map of 96 × 126 × 96 points with a distance spacing of 0.442 Angströms representing the three-dimensional protein structure was calculated and used for the docking. The appropriate atom-specific affinity maps were created. The grid map covered the entire protein structure so that the ligand could move freely within the grid around the protein. For the ligand, pamoic acid, flexible docking has been enabled by defining 8 rotatable bonds within the molecule. The search of the parameter space followed the Lamarckian genetic algorithm, with most of the user-defined parameters set to their default values. We modified the population size by setting it to 1000 individuals, as well as the number of surviving individuals, the maximum number of energy evaluations and the maximum number of generations, which were multiplied by 10 with respect to their default values. With the number of generated structures set to 256, a typical run averaged 6 hours on a single Xeon 3 GHz CPU. Run in parallel on a 40-nodes Linux cluster, with different seeds for random number generator, the above setup allowed obtaining 10240 ligand conformations each time the program was launched. The results have been analyzed with an in-house software, allowing the analysis of the intermolecular energy and atomic contacts.

## Authors' contributions

CH purified the protein; collected and processed NMR data, modelled, refined and analysed the complex structure. FB cloned the gene and supervised biochemical experiments. VG and MC processed and analysed NOESY data. VG supervised NMR analysis. OS set up the STD technique in the laboratory. JC performed docking experiments, Kd fitting and supervised molecular modeling. CC and AM initiated and coordinated the study. All authors read and approved the final manuscript.
